# Bedside Transcatheter Patent Ductus Arteriosus Device Occlusion in an Extremely Low Birth Weight Neonate: A Novel Approach in a High-Risk Population

**DOI:** 10.1155/2021/4716997

**Published:** 2021-10-27

**Authors:** Tiffany M. Pouldar, Robert Wong, Myriam Almeida-Jones, Evan Zahn, Lorraine Lubin

**Affiliations:** ^1^Department of Anesthesiology, Cedars-Sinai Medical Center, Los Angeles, CA, USA; ^2^Department of Pediatric Cardiology, Smidt Heart Institute and Department of Pediatrics, Cedars-Sinai Medical Center, Los Angeles, CA, USA; ^3^Guerin Family Congenital Heart Program, Smidt Heart Institute and Department of Pediatrics, Cedars-Sinai Medical Center, Los Angeles, CA, USA; ^4^Department of Anesthesiology, University of California, Los Angeles, CA, USA

## Abstract

Extremely low birth weight (ELBW) infants weighing less than 1 kilogram are at a high-risk for delayed patent ductus arteriosus (PDA) closure. Percutaneous PDA closure offers a less invasive approach when compared with surgical PDA closure, which may provide faster recovery times and less transfusion requirements. However, this procedure involves transporting tenuous, unstable patients from the neonatal intensive care unit (NICU) to the catheterization laboratory which introduces many potential risks for the neonate. Performing percutaneous PDA closure at the bedside offers a successful alternative to performing the procedure in the catheterization laboratory and avoiding risk associated with transporting ELBW neonates.

## 1. Introduction

Despite advances in healthcare technology and improvements in neonatal intensive care, extremely low birth weight (ELBW) infants weighing less than 1 kilogram remain at a high-risk for death and morbidity [1, 2]. Patients with patent ductus arteriosus (PDA) who do not respond to medical management often undergo surgical closure. With improved technology and equipment, there has been a shift to perform transcatheter PDA closure in ELBW infants as it has emerged as a safe and effective alternative to manage persistent PDA in preterm neonates [3]. Risk factors related to vascular access, device size, and overall medical fragility have been recently addressed with advances in the technique and newly available devices that are better suited for this population [3–5]. There are few reported cases of bedside percutaneous PDA closure as the procedure is usually completed in the catheterization laboratory. By combining the use of portable fluoroscopy and echocardiography, a bedside transvenous approach becomes a viable alternative to performing these procedures at the bedside, eliminating risks associated with transporting tenuous neonates [6, 7]. Written HIPAA authorization was obtained from the patient's legal guardian and the manuscript adheres to the EQUATOR and CARE guidelines.

## 2. Case Description

An 25-week-old infant was born via spontaneous vaginal delivery with a birth weight of 780 grams, requiring intubation shortly after birth secondary to respiratory failure. On day 6 of life, transthoracic echocardiogram (TTE) revealed a large PDA with unrestricted left-to-right shunting, 1.5 m/s peak velocity, and 9.2 mmHg peak gradient along with a patent foramen ovale and left-to-right shunting. Despite two courses of indomethacin, repeat TTE showed a moderate to large PDA with unchanged, unrestricted continuous left-to-right shunting.

At day 19 of life, weighing 790 grams, bedside percutaneous PDA closure under fluoroscopy and direct TTE imaging was performed. Standard American Society of Anesthesiology monitors were placed, with the addition of pre and postductal pulse oximetry to evaluate for aortic occlusion during device positioning and deployment. The anesthesiologist remained at the head of the bed with the ventilator positioned to the patient's right and TTE machine to the patient's left. The cardiologist performing the echocardiogram stood on the left side of the patient.

The infant was transferred to a special procedural bed (Rainbow Flex, NeoForce, Ivyland, PA). Prior to the procedure, she was induced with fentanyl 1 mcg/kg and cisatracurium 0.2 mg/kg and continued on fentanyl 6 mcg kg^−1^ hr^−1^. An umbilical arterial line previously placed on day 1 of life was used for blood pressure monitoring. She was continued on the neonatal intensive care unit (NICU) ventilator with pressure-controlled ventilation with peak inspiratory pressure of 19 cm H_2_0 and fractional inspired oxygen (FiO_2_) 30–40% for the duration of the procedure. Heparin was avoided (with the exception of heparinized saline within the catheters), since the patient had evidence of intraventricular hemorrhage in the first 10 days of life. The right femoral region was prepped and sterilely draped. A 4Fr introducer sheath was percutaneously placed into the right femoral vein using the standard Seldinger technique. A soft tipped guide wire was directed through the right heart, across the right ventricular outflow tract, down the PDA, and into the descending aorta using fluoroscopic guidance via a mini C-arm machine (Flouroscan InSight, Hologic, Bedford, MA). With the catheter passing across the tricuspid valve, a transient fall in blood pressure occurred presumably from transient stenting of the tricuspid valve resulting in regurgitation. Calcium chloride (10 mg/kg), saline fluid bolus (10 cc/kg), and dopamine (3 mg kg^−1^ min^−1^) were prepared ahead of time and primed in the infusion pumps. The patient did not require therapy as her transient hypotension resolved without intervention.

Under direct TTE imaging, a 3/2 mm Amplatzer Piccolo Occluder Device™ (Abbott Structural Heart, Plymouth, MN) was placed within the lumen of the PDA. Prior to release of the device from the delivery cable, a thorough imaging assessment was performed to evaluate device location, presence of residual ductal shunting, and evidence of left pulmonary artery and/or descending aorta obstruction. Following release of the device, TTE imaging confirmed device position, absence of residual shunt, and normal Doppler flow profiles within the left pulmonary artery and descending aorta.

The patient tolerated the procedure well, without perioperative complications. Though blood was crossmatched and readily available in the NICU, no transfusions were necessary secondary to the minimal blood loss. For the duration of the procedure, she remained on the NICU ventilator which provided adequate ventilation. She remained in sinus rhythm with systemic oxygen saturations >95% on 30–40% FiO_2_. Systolic arterial blood pressures remained above 70 mmHg and temperature was maintained above 37°C. Her postprocedure TTE showed stable device position slightly favoring the pulmonary artery aspect of the ductus arteriosus, without extension into the descending aorta or left pulmonary artery and without residual shunt across the ductus arteriosus.

## 3. Discussion

ELBW neonates who receive surgical PDA closure are at elevated perioperative risk including hypoperfusion secondary to decreased preload, increased systemic vascular resistance, and higher risk of bleeding [8]. Percutaneous PDA closure in ELBW patients offers a less invasive alternative to surgical PDA closure, with potentially less likelihood of hemodynamic instability and blood loss requiring transfusion [9]. Echocardiographic guided percutaneous closure at bedside or in the cardiac catheterization laboratory allows for the possibility of less (or no) contrast and radiation exposure [10]. Besides obtaining percutaneous femoral access, the procedure is not overly stimulating and usually requires, on average, 1 mcg/kg of fentanyl when compared to surgical PDA closure, which can require, on average, 10.5 mcg/kg of fentanyl for the duration of surgery [11].

In addition to the risks associated with the presence of a hemodynamically significant PDA, ELBW neonates can pose significant challenges to providers during transport, putting them at increased risk of complications and harm. Surgical PDA ligations on premature neonates were originally performed in the operating room. Multiple studies have shown safety and efficacy of a bedside procedure with equivalent rates of infection and mortality when compared to performing the procedure in the operating room. Some studies have demonstrated less hemodynamic changes when the procedure is completed at the bedside [12, 13]. ELBW infants are prone to hypothermia given their high surface area to bodyweight ratio and lack of subcutaneous fat, which can impair wound healing and result in coagulopathy. The risk of hypothermia can be mitigated by transporting the ELBW infants in a Giraffe isolette which provides active warming. However, transporting the Giraffe isolette can also be cumbersome and difficult as they can be large and difficult to maneuver [6]. ELBW infants are also at risk for accidental extubation and endotracheal tube (ETT) migration into a mainstem bronchus as simple movement of the head during transport can result in significant changes in ETT position. As inspired oxygen is a potent pulmonary vasodilator, the amount of FiO_2_ concentration must also be carefully titrated in these fragile infants so as to balance the shunt volume across the PDA while avoiding hyperoxia and hypoxia. Hypoxia-induced bradycardia is another significant risk in ELBW infants given the challenges of ventilating the fragile lungs that have experienced prolonged pulmonary over circulation along with decreased lung compliance secondary to pulmonary immaturity. Furthermore, transporting the ELBW infant can require many providers to concurrently manage the ventilator, move the isolette, and clear the path to the catheterization laboratory [6].

Performing the catheter-based PDA closure at the bedside in the NICU avoids or minimizes several of these challenges. Additionally, performing these procedures bedside facilitates use of NICU ventilators, as ELBW neonates have extremely small tidal volumes which most anesthesia machine ventilators are not able to accurately deliver given the increased dead space in the anesthesia circuit. Furthermore, if a neonate requires more complex ventilator support such as high frequency oscillatory ventilation, performing the procedure at the bedside eliminates the need for transporting, positioning, and setting up the high frequency oscillator in the catheterization laboratory.

Despite these many advantages, performing this procedure at the bedside (versus the catheterization lab) remains uncommon [12–14]. A lack of biplane fluoroscopy, limited imaging provided by mobile C-arms, and a lack of familiarity and comfort on the part of the interventional team are among the factors that contribute to this lack of widespread adoption of the bedside transcatheter procedure. Adequate monitoring should be available for anesthetizing the neonate at the bedside, such as capnography and IV equipment pumps for transfusions and medication administration.

This case report demonstrates that a large, hemodynamically significant PDA in an ELBW infant can be safely and effectively closed at the bedside in the NICU. Using a completely transvenous approach (mandatory in these tiny infants) is possible using a unique neonatal procedural bed, portable C-arm fluoroscopy, and standard TTE. While there is an understandable hesitancy on the part of most centers to adopt this technique, we anticipate that with refinements in equipment, technology, and technique as well as further experience, anesthesiologists will be performing more bedside PDA closures in ELBW neonates as it becomes the standard method of PDA closure in this unique patient population.

## Abbreviations

ELBW: Extremely low birth weight
PDA: Patent ductus arteriosus
NICU: Neonatal intensive care unit
TTE: Transthoracic echocardiogram
IRB: Institutional Review Board
FiO2: Fractional inspired oxygen.
